# The Alternatively Spliced Isoforms of Key Molecules in the cGAS-STING Signaling Pathway

**DOI:** 10.3389/fimmu.2021.771744

**Published:** 2021-11-18

**Authors:** Jiaqian Liang, Ze Hong, Boyue Sun, Zhaoxi Guo, Chen Wang, Juanjuan Zhu

**Affiliations:** ^1^ State Key Laboratory of Natural Medicines, School of Life Science and Technology, China Pharmaceutical University, Nanjing, China; ^2^ Department of Biomolecular Sciences, Weizmann Institute of Science, Rehovot, Israel

**Keywords:** alternative splicing, spliced isoforms, cGAS-STING signaling pathway, IFN response, antiviral response

## Abstract

Alternative splicing of pre-mRNA increases transcriptome and proteome diversity by generating distinct isoforms that encode functionally diverse proteins, thus affecting many biological processes, including innate immunity. cGAS-STING signaling pathway, whose key molecules also undergo alternative splicing, plays a crucial role in regulating innate immunity. Protein isoforms of key components in the cGAS-STING-TBK1-IRF3 axis have been detected in a variety of species. A chain of evidence showed that these protein isoforms exhibit distinct functions compared to their normal counterparts. The mentioned isoforms act as positive or negative modulators in interferon response *via* distinct mechanisms. Particularly, we highlight that alternative splicing serves a vital function for the host to avoid the overactivation of the cGAS-STING signaling pathway and that viruses can utilize alternative splicing to resist antiviral response by the host. These findings could provide insights for potential alternative splicing-targeting therapeutic applications.

## Introduction

**Figure 1:**
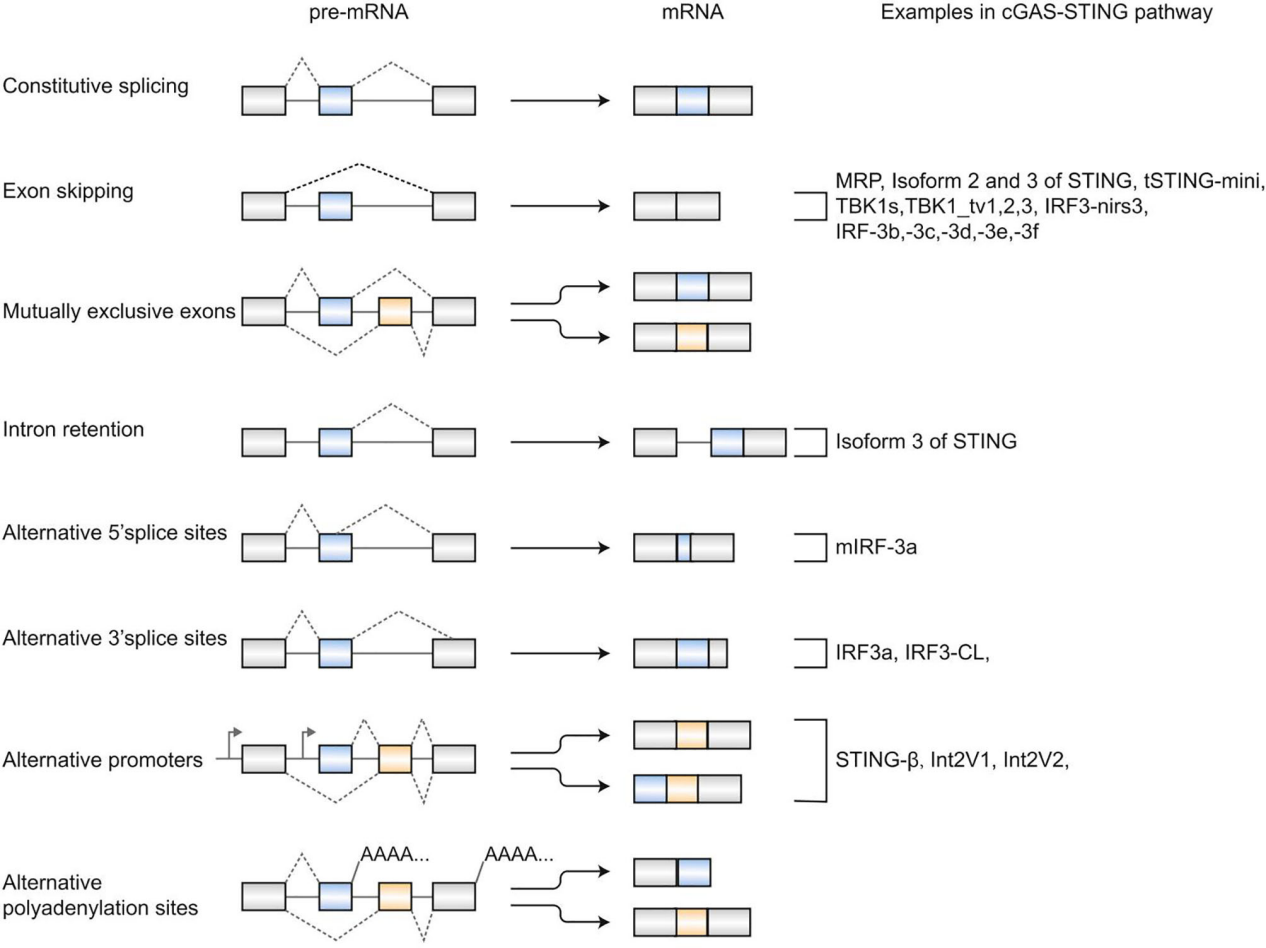
Patterns of alternative splicing. Exon skipping, mutually exclusive exons, intron retention, alternative 5’ splice sites, and alternative 3’ splice sites are five classical alternative splicing events. Alternative polyadenylation sites and alternative promoters also generate multiple mRNAs from a pre-mRNA. Alternative splicing- derived isoforms in the cGAS-STING signaling pathway are listed on the right.

In 1977, Chow et al. first reported alternative splicing (AS) as an essentially biological process in eukaryotes (1). Alternative splicing allows individual genes to generate numerous mRNA transcripts that encode proteins with similar or distinct functions. Typically, there are five major modes of alternative splicing events (2), including exon skipping, mutually exclusive exons, intron retention, alternative donor (5′ splice) sites, and alternative acceptor (3′ splice) sites (**Figure 1**). The most frequent event of alternative splicing is exon skipping, while mutually exclusive exons are an uncommon subtype. In addition to these five alternative splicing modes, alternative polyadenylation sites and alternative promoters also contribute to proteome complexity (3–5), although they are indirectly related to splicing (**Figure 1**). Previous studies have reported that alternative splicing is involved in expressing >95% of human genes (6, 7). Various physiological processes have been reported to be related to alternative splicing, such as apoptosis (8), sex determination (9), T-cell activation (10, 11), and DNA repair (12). Notably, misregulation of alternative splicing is related to a series of diseases, including Huntington’s disease (13), diabetes mellitus (14), and Alzheimer’s disease (15). Thereby, a better knowledge of alternative splicing is critical for treating diseases involving splicing.

Innate immunity is the initial line of defense against pathogen invasion, whose activation necessitates the detection of pathogens *via* pattern recognition receptors (PRR) (16). PRR recognizes pathogen-associated molecular patterns (PAMP) (17) and further activates the expression of type I IFN (IFN-I) and pro-inflammatory cytokines. Upon viral infection, the virus nucleic acid acts as a PAMP to trigger antiviral immune responses (18). Cyclic GMP-AMP synthase (cGAS) is a cytoplasmic PRR that recognizes and binds with double-stranded DNA (19, 20). The stimulator of interferon genes (STING), an adaptor protein downstream of cGAS, is one of the most important molecules involved in the antiviral innate immune response (21–23). During infection with DNA virus, cGAS senses viral DNA and catalyzes the synthesis of cGAMP from ATP and GTP. As a second messenger, cGAMP then binds to STING. STING translocates from the ER to the Golgi apparatus upon ligand binding, where STING recruits TBK1 to activate IRF3. The activated IRF3 further dimerizes and shuttles to the nucleus to initiate the transcriptional expression of IFN-I and IFN-stimulated genes (ISGs). While the cGAS-STING signaling pathway is critical for DNA virus-triggered antivirus response, retinoic acid-inducible gene (RIG)-I-like receptors (RLRs), such as retinoic acid-induced gene-I (RIG-I) and melanoma differentiation-associated gene 5 (MDA5), are primarily responsible for activating TBK1-IRF3-mediated IFN response by sensing viral RNA (24).

The cGAS-STING-mediated IFN-I signaling pathway is a significant discovery in the field of innate immunity, especially its indispensable role in antiviral response. However, excessive activation of the cGAS-STING signaling pathway results in autoimmune diseases, such as STING-associated vasculopathy with onset in infancy (SAVI) (25), lupus-like disease (26), and Ataxia-telangiectasia (AT) (27). Increasing evidence shows that alternative splicing is involved in the precise regulation of innate immunity (28). This review summarizes the alternatively spliced isoforms discovered in the cGAS-STING-TBK1-IRF3 axis. It elucidates their functions in antiviral response, autoimmune diseases and cancer, thus obtaining a better understanding of the regulatory role of alternative splicing in the cGAS-STING signaling pathway and innate immunity.

## Alternatively Spliced Isoforms in the cGAS-STING Signaling Pathway

Alternative splicing results to diversiform mRNAs and proteins in eukaryotes. Recently, numerous alternatively spliced isoforms of cGAS, STING, TBK1, and IRF3 have been found in human, mouse, Chinese tree shrew or zebrafish. The following will describe the alternatively spliced isoforms that have been identified in the cGAS-STING signaling pathway to date (**Table 1**).

**Table 1:** Alternatively spliced isoforms that have been identified in the cGAS-STING signaling pathway.

Counterparts	Isoforms	Splice form	Modle/System	References
cGAS	cGAS isoforms	skip exon 2, 3, 4, 5 or a combination of them	hominoid, Old World, and New World Monkey species	(29)
STING	MRP	lacks exon 7	various human tissues and cell lines	(30, 31)
	Isoform 2	lacks exon 7 and exon 4	PBLs, liver biopsy specimens	
	Isoform 3	lacks exon 7, and contains an unspliced intron after exon 3		
	STING-β	uses alternative transcription start sites in intron 5	various human tissues and cell lines	(32)
	tSTING-mini	skips exons 2-5	Chinese tree shrew	(33)
TBK1	TBK1s	lacks exons 3-6	mouse and human cells	(34)
	TBK1_tv1	lacks exon 3 and 4	zebrafish	(35)
	TBK1_tv2	excises exons 4-18		(36)
	TBK1_tv3	lacks exons 14-17		
IRF3	IRF3a	uses alternative 3’ splice sites of IRF-3 pre-mRNA	majority of tissues and cell lines	(37, 38)
	IRF3-nirs3	skips exon 6	hepatocellular carcinoma	(39)
	IRF3-CL	uses an alternative 3’ spliced site at the upstream of exon 7	normal Chang liver cell line and tumor cell lines	(40)
	IRF-3b(3d)	excises exon 2, 3, 6, or a combination of them	various human cells and tissues	(41)
	IRF-3c(3f)			
	IRF-3e			
	mIRF-3a	uses alternative 5’ splice sites within exon 6	mouse tissues	(42)

Currently, there are few studies about cGAS isoforms. Hancks et al. (2015) cloned multiple alternatively spliced isoforms of cGAS from hominoid, Old World, and New World Monkey species (29). The identified isoforms were generated from exon skipping. Although few studies on the structural and functional characterization of cGAS isoforms, we can speculate their function based on domain composition. The full-length cGAS mRNA contains 5 exons. Due to the presence of exon 1, all the identified spliced forms of cGAS may contain intact DNA binding domain and may retain DNA binding activity. The isoforms that lack exon 3 but maintain exon 2 are likely to contain cGAMP catalytic residues still, leading to the potential ability to synthesize cGAMP. However, these cGAS spliced isoforms may provide an escape means for pathogen owing to the loss of several sites under positive selection in exon 3. Nevertheless, these conjectures need further research to confirm.

Contrary to rarely reported cGAS isoforms, the *STING* gene processes numerous alternatively spliced isoforms. In human cells, three alternatively spliced isoforms of STING mRNA that lack exon 7 compared with canonical mRNA are collectively called E7-less hSTING isoforms, which are MRP (MITA related protein), truncated isoform 2 and 3 of STING (30, 31). In addition, isoform 2 also lacks exon 4, whereas isoform 3 retains an unspliced intron after exon 3. These three proteins all lack the C-terminal domain (CTD) essential for recruiting TBK1 and IRF3. A shortened STING isoform has also been described in the Chinese tree shrew (Tupaia belangeri chinensis) in 2020, termed tSTING-mini (33). The mRNA encoding tSTING-mini is transcribed by exon skipping, and the encoded protein shares the first three TM regions with tSTING-FL (a full-length Tupaia STING).

TBK1 is also subject to alternative splicing. The first alternatively spliced isoform of TBK1, denoted as TBK1s, was identified in mammals in 2008 (34). TBK1s mRNA lacks exons 3-6 compared with TBK1 mRNA, resulting in an alternative translation start site at the second ATG and the loss of kinase domain (KD) in TBK1s protein. In addition to in mammals, three alternatively spliced isoforms of TBK1 also generated by exon skipping have been discovered in zebrafish in the last 3 years, namely TBK1_tv1, TBK1_tv2, and TBK1_tv3, respectively (35, 36). Compared with TBK1, TBK1_tv1 and TBK1_tv2 lack the complete kinase domain due to exon deletions.

IRF3 isoforms were first discovered as early as 20 years ago, and many IRF3 isoforms have been identified since then. IRF-3a, the first identified spliced isoform of human IRF3 in 2000, is produced using alternative 3′ splice sites of hIRF-3 pre-mRNA (37). As a result, IRF-3a protein contains an incomplete DNA binding domain (DBD) at the N-terminus. IRF-3b, -3c, -3d, -3e, and -3f are five spliced isoforms of IRF3 that result from the excision of single exons (like exons 2, 3, or 6) or a combination of them (41). Except for IRF-3e, the other four isoforms all lack complete DBD. In hepatocellular carcinoma (HCC) cells, IRF3-nirs3 mRNA is generated by skipping of exon 6, and the translated protein lacks 127 amino acids contained in the IRF association domain (IAD), which is crucial for the activation of IRF3 (39). IRF3-CL lacks IAD and the C-terminal autoinhibition element (AIE) due to an alternative 3’ spliced site upstream of exon 7 (40). In addition, mIRF-3a lacks partial N-terminal IAD due to the utilization of alternative 5′ splice sites within exon 6 of mouse IRF3 mRNA (42).

Most of the STING, TBK1, IRF3 isoforms are generated by exon skipping (**Figure 1**) consistent with exon skipping being the most frequent alternative splicing event. In addition to the five classical alternative splicing events, alternative polyadenylation sites and alternative promoters also expand the variety of mRNAs and proteins. STING-β, another alternatively spliced isoform of STING, is generated by using alternative transcription start sites distinct from hSTING. STING-β contains 25 unique amino acids at the N-terminus and shares the functional CTD with hSTING (32). Moreover, two novel IRF-3 transcripts transcribed from two new transcriptional start sites were found in pheochromocytoma cells, namely Int2V1 and Int2V2, respectively (43). However, more studies are needed to characterize the structural composition of Int2V1 and Int2V2. As a gene may produce multiple or even thousands of transcripts through different splicing patterns, more isoforms might be waiting to be discovered in the cGAS-STING signaling pathway.

## Regulation of cGAS-STING Signaling Pathway by Alternative Splicing

**Figure 2:**
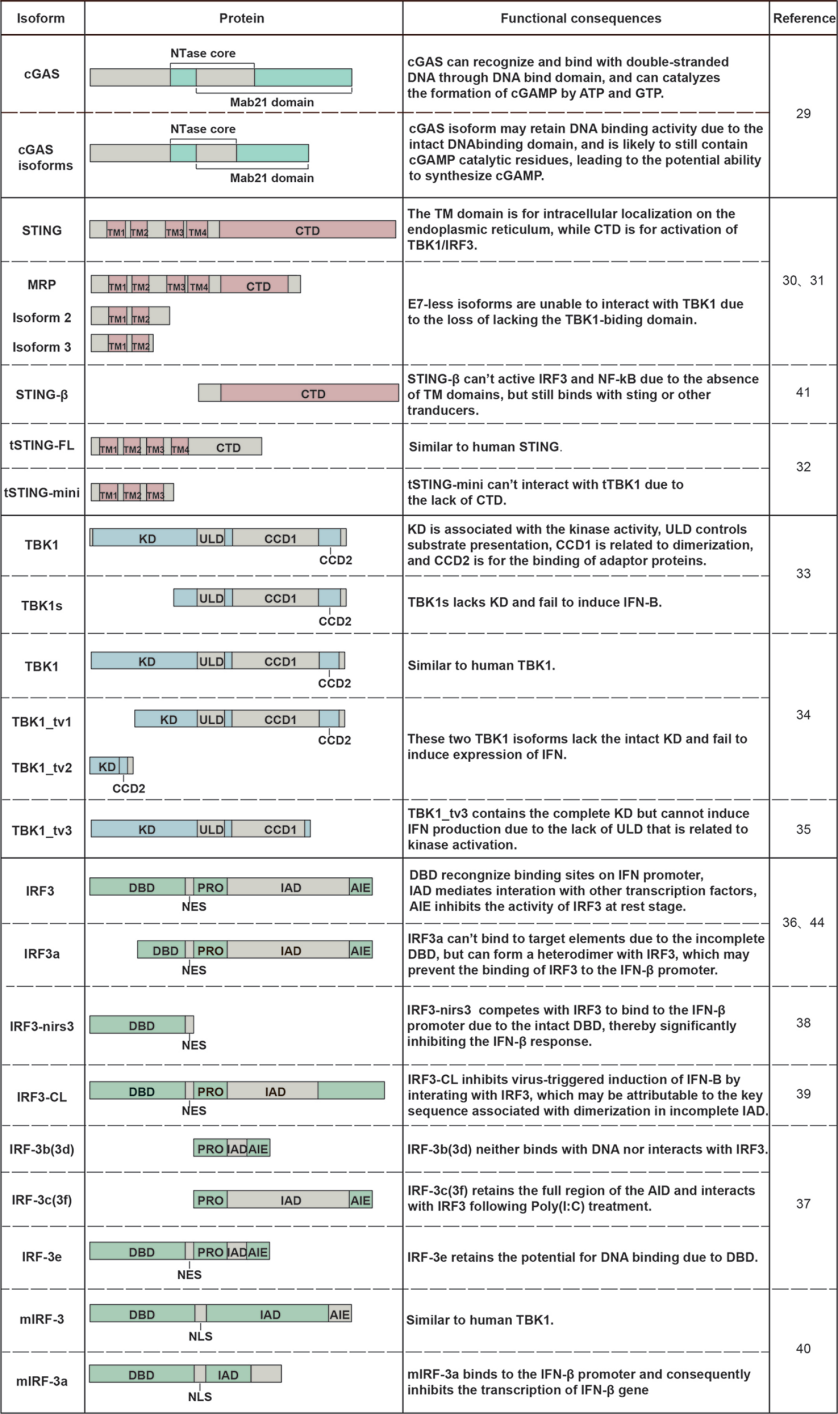
Changes in the domain composition of spliced isoforms and their functional consequences. The difference in domain composition between STING/TBK1/IRF3 alternatively spliced isoforms and their counterparts and consequent functional changes are shown in the figure.

**Figure 3:**
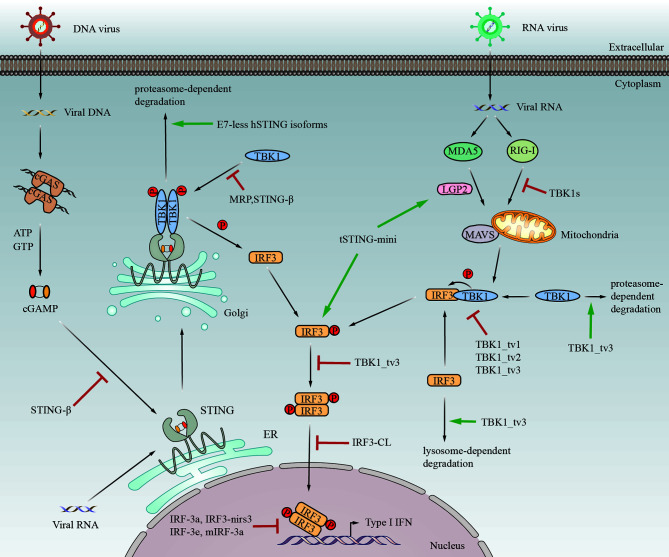
The immunological functions of STING/TBK1/IRF3 alternatively spliced isoforms in response to viral infection. tSTING-mini induces antiviral response after RNA virus infection by binding to tMDA5-tLGP2. Moreover, tSTING-mini strongly interacts with tIRF3 and promotes phosphorylation of tIRF3 without tTBK1. Except for tSTING-mini, the other spliced isoforms displayed in the figure negatively regulate IFN response by distinct mechanisms. E7-less hSTING isoforms promote the degradation of STING through the proteasome pathway. MRP also inhibits IFN response by disrupting the STING-TBK1 interaction. STING-β can block the interaction between STING and cGAMP or TBK1. TBK1s binds to RIG-I to block the interaction between RIG-I and MAVS upon Sendai virus infection. TBK1_tv1, TBK1_tv2 and TBK1_tv3 disrupt the formation of TBK1-IRF3 complexes by competitively binding to TBK1 and IRF3. In addition, TBK1_tv3 induces TBK1 and IRF3 degradation mediated by the ubiquitin-proteasome pathway and lysosome pathway, respectively. Moreover, IRF3a can form a heterodimer with IRF3, which may prevent the binding of IRF3 to the IFN-β promoter. IRF-3e maintains the ability to bind to DNA and compete with IRF3 for binding to the IFN-β promoter. IRF3-nirs3 competes with IRF3 to bind to the IFN-β promoter sequence. IRF3-CL inhibits the shuttling of IRF3 from the cytoplasm to the nucleus. mIRF-3a binds to the IFN-β promoter and consequently inhibits the transcription of the IFN-β gene. The mechanism for IRF-3c (3f) to reduce the transactivation of the IFN-β promoter remains unclear. Additionally, IRF-3b (3d) has no significant effect on IFN response.

Alternative splicing generates protein isoforms with different domain compositions. The gain and loss of protein domains lead to potential functional changes, including subcellular localization, binding capacity, and enzymatic activity (44) (**Figure 2**). A single gene can generate multiple protein isoforms with diverse functions that may be similar to or even antagonistic to their normal counterparts. What is more, the isoforms mentioned above may function as negative regulators or positive effectors in innate immunity (**Figure 3**).

### Negative Regulation

Almost all the identified isoforms in the cGAS-STING signaling pathway play a negative regulatory role in IFN response. STING is responsible for the induction of type I IFN against pathogen invasion. However, the E7-less STING isoforms (isoform 2, isoform 3, and MRP) failed to induce the expression of IFN-β and even negatively regulated STING-mediated IFN response *via* reducing the stability of STING through proteasome-dependent degradation, which was facilitated by the direct interaction between E7-less isoforms and STING (31). Moreover, MRP also inhibits IFN response by blocking the connection between STING and TBK1. Wang et al. have demonstrated the inhibitory effect of endogenous STING-β on the expression of type I IFN and ISGs induced by cyclic dinucleotides, DNA, RNA, and viruses (32). Mechanistically, STING-β disrupts the interaction between STING and transducers to block the activation of downstream signal transduction.

The isoforms of TBK1 have inhibitory effect on IFN response. Deng et al. (2008) showed that the overexpression of TBK1s down-regulated SeV triggered-IFN-β production by blocking the interaction between RIG-I and MAVS and by disrupting the cytoplasm-to-nucleus shuttling of IRF3 (34). Moreover, although it lacks the kinase domain, TBK1s may compete with TBK1 to bind to IRF3. TBK1 isoforms identified in Zebrafish have been reported to act as inhibitory of IFN-I response and ISG expression against spring viremia of carp virus (SVCV) infection through distinct mechanisms (35, 36). TBK1_tv1, TBK1_tv2, and TBK1_tv3 disrupt the formation of TBK1-IRF3 complexes by competitively binding to TBK1 or IRF3, thereby effectively inhibiting the production of IFN. In addition, TBK1_tv3 induces IRF3 and TBK1 degradation *via* the lysosome pathway and ubiquitin-proteasome pathway, respectively.

The activity of IRF3 is also negatively regulated by its spliced isoforms, which will finally affect the activation of the IFN-β promoter. Usually, after being phosphorylated by TBK1, two molecules of IRF3 will form a dimer and transfer into the nucleus, binding to and transactivating the target promoter. The binding of IRF3 to the IFN-β promoter is always disrupted by the spliced isoforms of IRF3, including human IRF-3a, mouse IRF-3a, and IRF3-nirs3 (38, 39, 42). Research demonstrated that IRF-3e and IRF-3c (3f) possess the ability to diminish the transactivation of IFN-β promoter to disparate degrees, while IRF-3b (3d) have no impact on it (41). Upon VSV infection, the overexpression of IRF3-nirs3 downregulates the activity of IRF3 and competes with IRF3 to bind to the IFN-β promoter sequence, thereby significantly inhibiting the IFN-β response and making hepatocellular carcinoma (HCC) more susceptive to viral infection (39). Moreover, overexpression of IRF3-CL negatively regulates SeV-induced IFN-β response and inhibits the shuttling of IRF3 from the cytoplasm to the nucleus (40).

### Positive Regulation

In addition to the negative regulation, the alternatively spliced isoforms in the cGAS-STING signaling pathway, namely tSTING-mini and tSTING-FL, play a positive regulatory role in IFN response. tSTING-FL retains its classic function of resisting DNA virus infection, while tSTING-mini is essential for RNA virus-triggered antiviral response. Unlike the STING isoforms described above, tSTING-mini positively regulates and enhances the production of IFN-I (33). Mechanistically, tSTING-mini connects with tMDA5-tLGP2 in the early stage. In addition, tSTING-mini strongly interacts with tIRF3 and promotes phosphorylation of tIRF3 without recruiting tTBK1, thus enhancing the antiviral response. Most importantly, tSTING-mini induces quicker and more effective antiviral signaling than tSTING-FL upon RNA virus infection.

Although STING, TBK1, and IRF3 are essential for activating the IFN gene, their isoforms usually act as negative regulators in the IFN response by distinct mechanisms, which may be conducive to preventing the overactivation of the cGAS-STING signaling pathway. The spliced isoforms exhibit functions opposite to those of their normal counterparts, implying the vital role of alternative splicing in fine-tuning molecular signal transduction.

## Alternatively Spliced Isoforms of the cGAS-STING Signaling Pathway in Viral Infections and Diseases

Alternative splicing is critically required for diverse physiological and pathological processes and is crucial in maintaining cell homeostasis. Dysregulation of alternative splicing will lead to gene dysfunction, protein expression, function disorder, and even severe diseases. Below we will discuss the research advances of STING/TBK1/IRF3 isoforms in viral infections, systemic lupus erythematosus (SLE), and cancer.

### Viral Infection

Overexpression of most spliced isoforms would inhibit IFN production and host antiviral response, including IRF3-CL, STING-β, and so on. On the condition of SVCV infection, overexpression of TBK1_tv1 and TBK1_tv2 hampers the production of IFN-β mediated by RIG-I, MAVS, TBK1, and IRF3 (35). When IRF3-CL was highly expressed, it would reduce the induction of IFN-β triggered by SeV (40). The high expression of IRF3-nirs3 in IFN-β-competent cells increased virus replication (39). Under VSV or HSV infection, overexpression of STING-β enhanced viral replication and inhibited STING-mediated antiviral response (32). The evidence above suggests that these isoforms have the potential effect to enhance viral infection. Notably, unlike the isoforms imposing inhibitory effect, the overexpression of tSTING-mini enhances the expression of IFN-β activated by NDV and SeV and reduces viral replication of VSV (33).

Remarkably, alternative splicing serves as a strategy for viruses to escape antiviral response. Different viruses induce the fall or rise of isoform expression. Research showed that the ratio of STING to E7-less isoforms decreased during viral infection (31). Moreover, on the knock-down of E7-less isoforms, stronger STING activation was observed after viral infection, leading to reduced viral replication. Therefore, viruses may utilize E7-less isoforms to evade antiviral responses. MRP expression was increased in HSV-1 infection but decreased upon SeV invading, indicating that DNA virus and RNA virus might lead to differential expression of MRP (30). In addition, IRF-3 was degraded after 9 h post-viral infection, while the protein level of IRF3a did not change significantly (38). The increased IRF3a/IRF3 after viral infection may be a mechanism employed by virus to weaken the IFN response. Studies have shown that a higher expression of TBK1s is detected in HCV-infected patients than in healthy samples, implying that TBK1s may be involved in HCV infection (34). These findings together indicate that viruses can up-regulate the expression of spliced proteins conducive to their survival to counteract antiviral response.

### Systemic Lupus Erythematosus

Systemic lupus erythematosus (SLE) is an inflammatory autoimmune disease distinguished by an abnormal expression level of IFN-I (45). There is continuous evidence to prove the pathogenic role of the cGAS-STING signaling pathway in SLE. Compared with healthy people, the cGAS-STING signaling pathway is more active in SLE patients’ monocytes (46). Notably, the low plasma level of STING-β transcript was found in SLE patients. Considering the dominant-negative effect in STING-mediated IFN response, STING-β may play an inhibitory role in the hyperactivation of IFN signaling that potentially leads to autoimmune disease. Therefore, STING-β can be exploited as a therapeutic target for SLE.

### Cancer

Splicing perturbations normally occur in tumors, and tumor cells possess the ability to up-regulate the expression of advantageous protein by interfering with the host’s alternative splicing system (47). IRF-3b, -3c, -3d, -3e, and -3f are expressed more frequently in esophageal and liver tumor tissues than in normal tissues, suggesting that spliced isoforms may express in a tumor-specific manner. Moreover, the expression level of IRF3-nirs3 in hepatocellular carcinoma (HCC) is much higher than that in primary human hepatocytes (PHHs). This evidence implies that IRF-3(b-f) and IRF3-nirs3 have a potential role in the etiopathogenesis of cancer. What is more, splicing alterations can function as biomarkers for tumor diagnosis (48). Currently, small molecules with splicing regulatory function, antisense oligonucleotides that can specifically bind to pre-mRNA, and CRISPR-based approaches have been designed to interfere with the alternative splicing processes in cancer cells (49), thereby producing cytotoxicity and killing tumor cells. Therefore, IRF-3(b-f) and IRF3-nirs3 may be recognized as potential markers for cancer diagnosis and may serve as targets to develop ideal treatment methods.

Overall, a certain spliced isoform greatly connects with a particular disease. Abnormal expression of spliced isomers found in diseases can be recognized as markers for diagnosis and may become therapeutic targets.

## Regulation of Alternative Splicing and Drug Discovery

Alternative splicing is a highly controlled process involving many regulatory components, mainly cis-acting elements and trans-acting factors. Splicing factors are recruited by cis-acting elements to promote or suppress the recognition of nearby splice sites, which are cleaved and linked by the spliceosome. Many disease-causing mutations in introns and exons affect the recognition of cis-acting elements by spliceosome (50). Moreover, alterations in spliceosomes would affect the activity of splicing machinery, which induces the occurrence of diseases (51). Therefore, ingredients involved in the splicing regulation can be targeted for therapeutic development, and numerous drugs are designed to correct erroneous splicing events. For example, the defects in the expression of survival of motor neuron (SMN) protein, which is encoded by the *SMN1* gene, will give rise to spinal muscular atrophy (SMA). Spinraza, an antisense oligonucleotide (ASO) that increases the expression of SMN proteins by enhancing exon 7 splicing through complementary binding to the *SMN2* gene, has been approved by FDA in 2016 (52). There are few studies about the splicing regulation of cGAS, STING, TBK1, and IRF3 isoforms. These isoforms generally express much lower than their counterparts in the resting state, but their expressions are abnormal in diseases or cancers. Furthermore, the underlying regulatory mechanisms remain unknown. Whether these isoforms can be utilized as therapeutic targets needs further investigation.

## Conclusion

Alternative splicing is an important regulatory mechanism in innate immunity, and spliced isoforms have been discovered in multiple immunoregulatory proteins. Here we reviewed the alternatively spliced isoforms of key molecules in the cGAS-STING signaling pathway, as well as their functions. Although cGAS, STING, TBK1, and IRF3 promote the expression of type I IFN, most of their spliced isoforms exhibit antagonistic functions relative to their normal counterparts. The mentioned isoforms act as positive or negative modulators in IFN response *via* distinct mechanisms. Particularly, we demonstrate that alternative splicing plays a pivotal role in avoiding the overactivation of the cGAS-STING signaling pathway, and that viruses can utilize alternative splicing to resist antiviral response by the host. Furthermore, alternative spliced isoforms can also provide therapeutic tactics for diseases including cancers. Alternative splicing events may even have the potential to become biomarkers to diagnose tumors due to their abnormal expression. However, plenty of spliced isoforms of immune-related genes need to be revealed, and the functions of known isoforms are still not fully understood. Therefore, much work needs to be done to depict further the regulatory mechanism of alternative splicing in the immune system.

## Author Contributions

JL and JZ conceived and wrote the manuscript. ZH, BS, and ZG processed the figures. CW and JZ organized the manuscript. All authors contributed to the article and approved the submitted version.

## Funding

The following grants supported this work, National Natural Science Foundation of China (No. 31730018) and Project Program of State Key Laboratory of Natural Medicines, China Pharmaceutical University (No. SKLNMZZ202002).

## Conflict of Interest

The authors declare that the research was conducted in the absence of any commercial or financial relationships that could be construed as a potential conflict of interest.

## Publisher’s Note

All claims expressed in this article are solely those of the authors and do not necessarily represent those of their affiliated organizations, or those of the publisher, the editors and the reviewers. Any product that may be evaluated in this article, or claim that may be made by its manufacturer, is not guaranteed or endorsed by the publisher.
